# Supplementary material for: Protein lipoylation in mitochondria requires Fe–S cluster assembly factors NFU4 and NFU5
Source: Plant Physiol. 2021 Oct 28;188(2):997–1013. doi: 10.1093/plphys/kiab501 (PMC8825329; doi:10.1093/plphys/kiab501)
Supplement: kiab501_Supplementary_Data [file kiab501_supplementary_data.pdf]

SUPPLEMENTAL DATA

Protein lipoylation in mitochondria requires Fe-S cluster assembly factors NFU4 and NFU5.

Przybyla-Toscano J, Maclean AE, Franceschetti M, Liebsch D, Vignols F, Keech O, Rouhier N and Balk J.

Supplemental Figure S1. NFU4 and NFU5 are localized in the mitochondrial matrix.

Supplemental Figure S2. Quantification of NFU5 protein levels from the *nfu5-2* allele.

Supplemental Figure S3. Root elongation in seedlings grown on vertical plates.

Supplemental Figure S4. Characterization of the *isu1-1* mutant allele alone and combined with *nfu4-2*.

Supplemental Figure S5. Protein blot and BN-PAGE analysis of mitochondria from *nfu4* and *nfu5* single mutants.

Supplemental Figure S6. Growth of *nfu4-2 nfu5* mutants under low CO_2.

Supplemental Figure S7. Elevated CO_2 cannot rescue growth of *nfu4 nfu5* mutants to produce seeds.

Supplemental Figure S8. Protein blot analysis of lipoylated proteins.

Supplemental Figure S9. Bimolecular fluorescence complementation to test interaction between LIP1 and NFU4/5, continued.

Supplemental Figure S10. Lipoic acid in growth medium does not rescue the *nfu4 nfu5* mutant phenotype.

Supplemental Table S1. *isu1*, *nfu4* and *nfu5* T-DNA insertion lines used in this study.

Supplemental Table S2. Free, water-soluble amino acid concentrations in *nfu4-2 nfu5-1* seedlings and wild-type like segregants.

Supplemental Table S3. Organic acid concentrations in wild-type, nfu4-2 nfu5-1 and wild-type like segregants.

Supplemental Table S4. Primers used in this study.

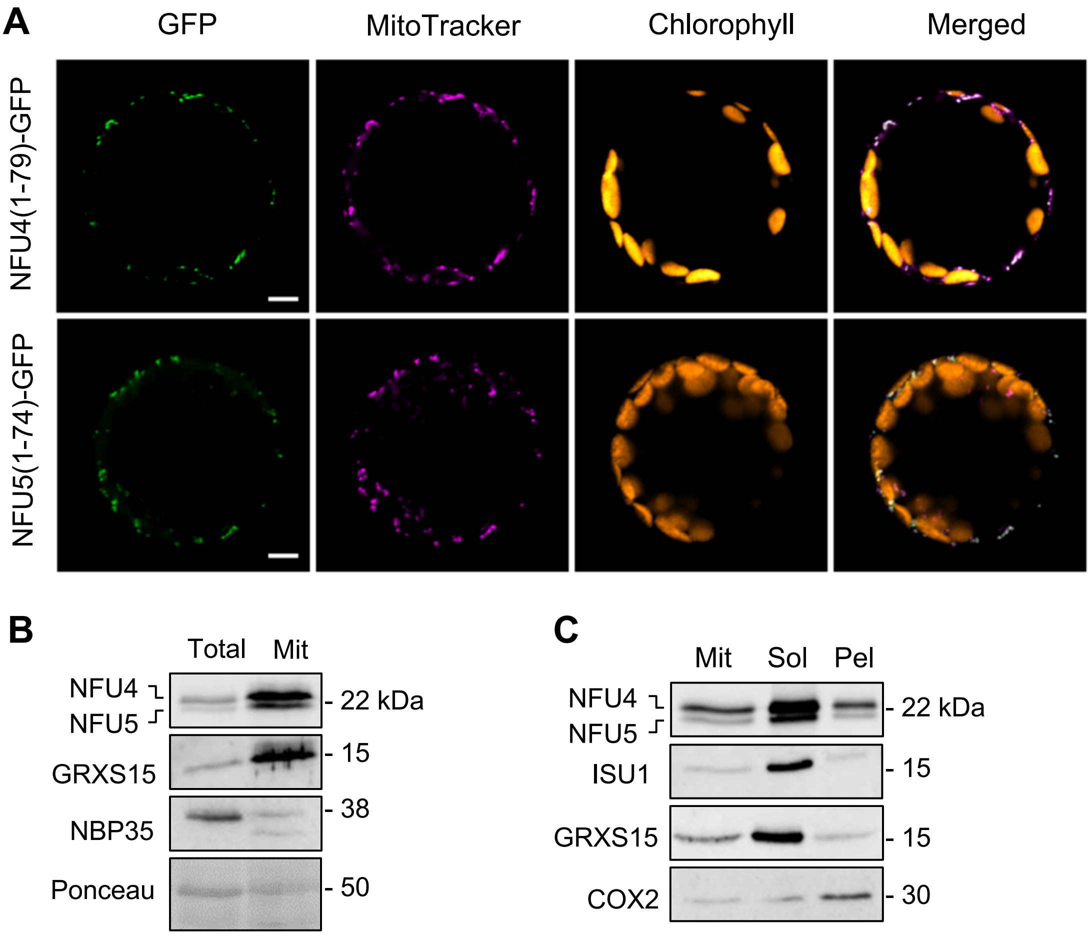

Supplemental Figure S1. NFU4 and NFU5 are localized in the mitochondrial matrix.
A. Transient expression of the N-terminal sequence of *NFU4*, encoding amino acids 1 – 79, fused to GFP (NFU4(1-79)-GFP) and similar for *NFU5* (NFU5(1-74)-GFP), under the control of a double *CaMV* 35S promoter, in Arabidopsis protoplasts. GFP signal in green. Cells were stained with MitoTracker Orange CMTMRos to show mitochondria (magenta). Autofluorescence of chlorophyll identifies the chloroplasts (orange). Scale bars: 5 µm.
B, C. NFU4 and NFU5 are enriched in mitochondria (B) and reside in the soluble matrix fraction (C). Protein blot analysis of total leaf extract and purified mitochondria (Mit) from wild-type Arabidopsis seedlings. The mitochondria were separated into soluble matrix proteins (Sol) and the pellet (Pel) enriched in membrane proteins following 10-min centrifugation at 16,000 x *g*. Protein blots were labelled with antibodies against NFU4, which cross-react with both NFU4 and NFU5. ISU1 and GRXS15 were previously shown to be soluble matrix proteins and COX2 served as a membrane marker protein. The apparent molecular weights in kDa are indicated on the right.

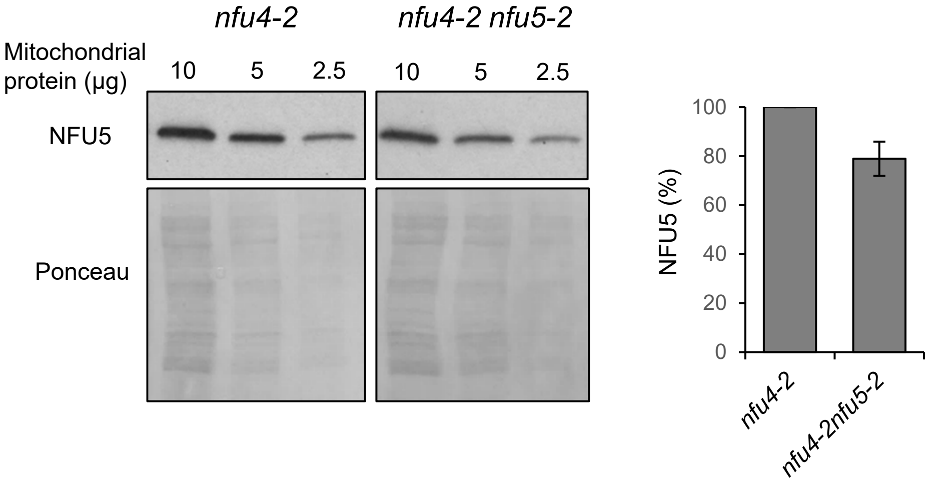

Supplemental Figure S2. Quantification of NFU5 protein levels from the *nfu5-2* allele. Leaf protein extracts from an *nfu4-2 nfu5-2* double mutant were compared with *nfu4-2* to avoid interference from the NFU4 immune signal. Signals were quantified by densitometry using ImageJ software. Error bar represents SD, n=3.

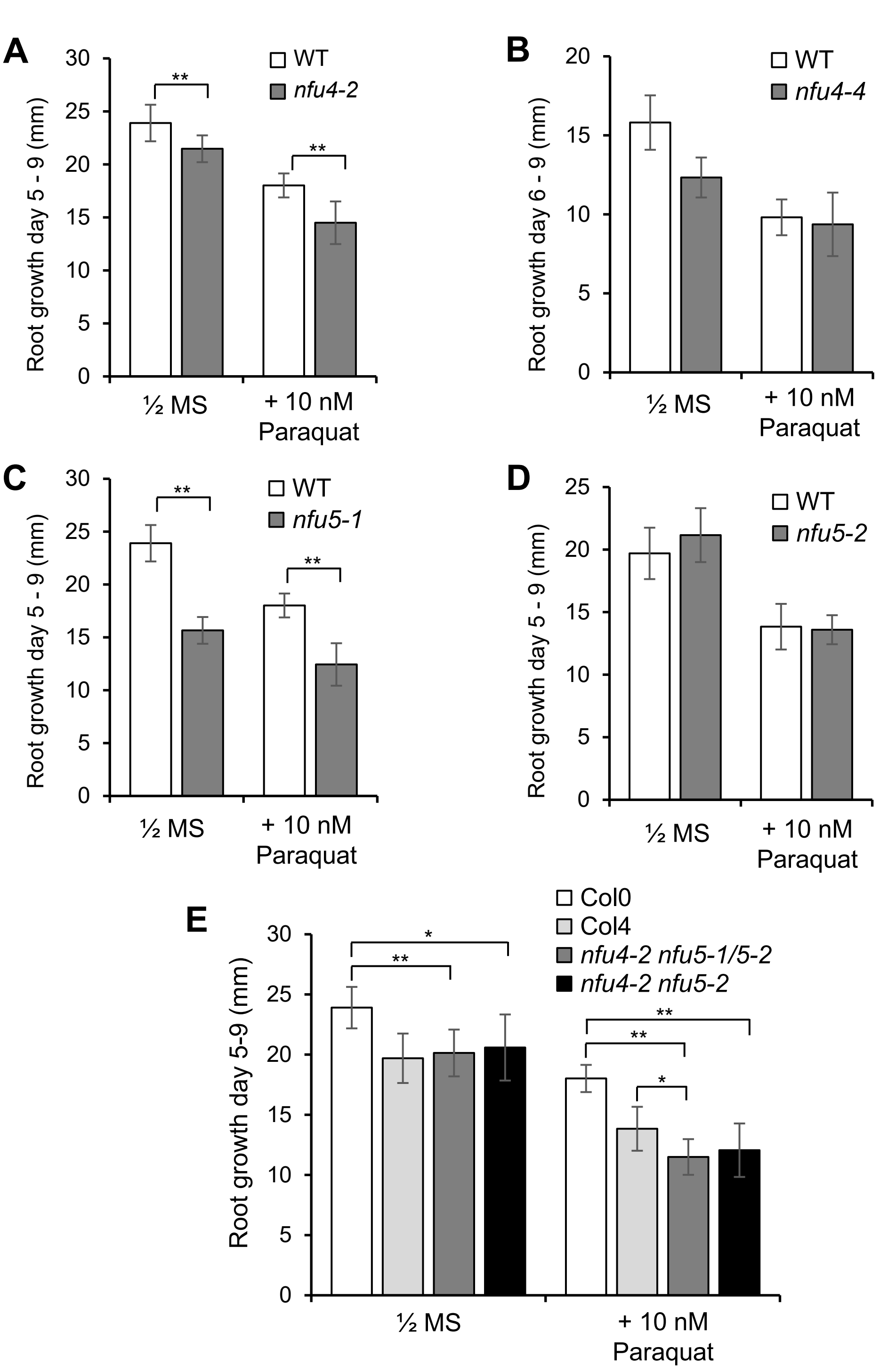

Supplemental Figure S3. **Root elongation in seedlings grown on vertical plates.**
Seedlings were grown on half-strength Murashige & Skoog (½ MS) medium for 5 days, then transferred to fresh plates without or with 10 nM Paraquat, an inducer of reactive oxygen species in mitochondria, and grown for another 3 - 4 days. Values represent the average elongation from day 5 to day 9 (day 6 – 9 for *nfu4-4*), error bars represent SD.
A. *nfu4-2* (Col-0); **B** *nfu4-4* (Col-0); **C.** *nfu5-1* (Col-0); **D.** *nfu5-2* (Col-4); **E.** The double mutants *nfu4-2 nfu5-1/5-2* and *nfu4-2 nfu5-2*. The respective wild-type lines are indicated in brackets. *p<0.01, ** p<0.001 (Student *t*-test) , n=30-31 for Col-0, n=12-13 for Col-4, n>9 for all mutant lines.

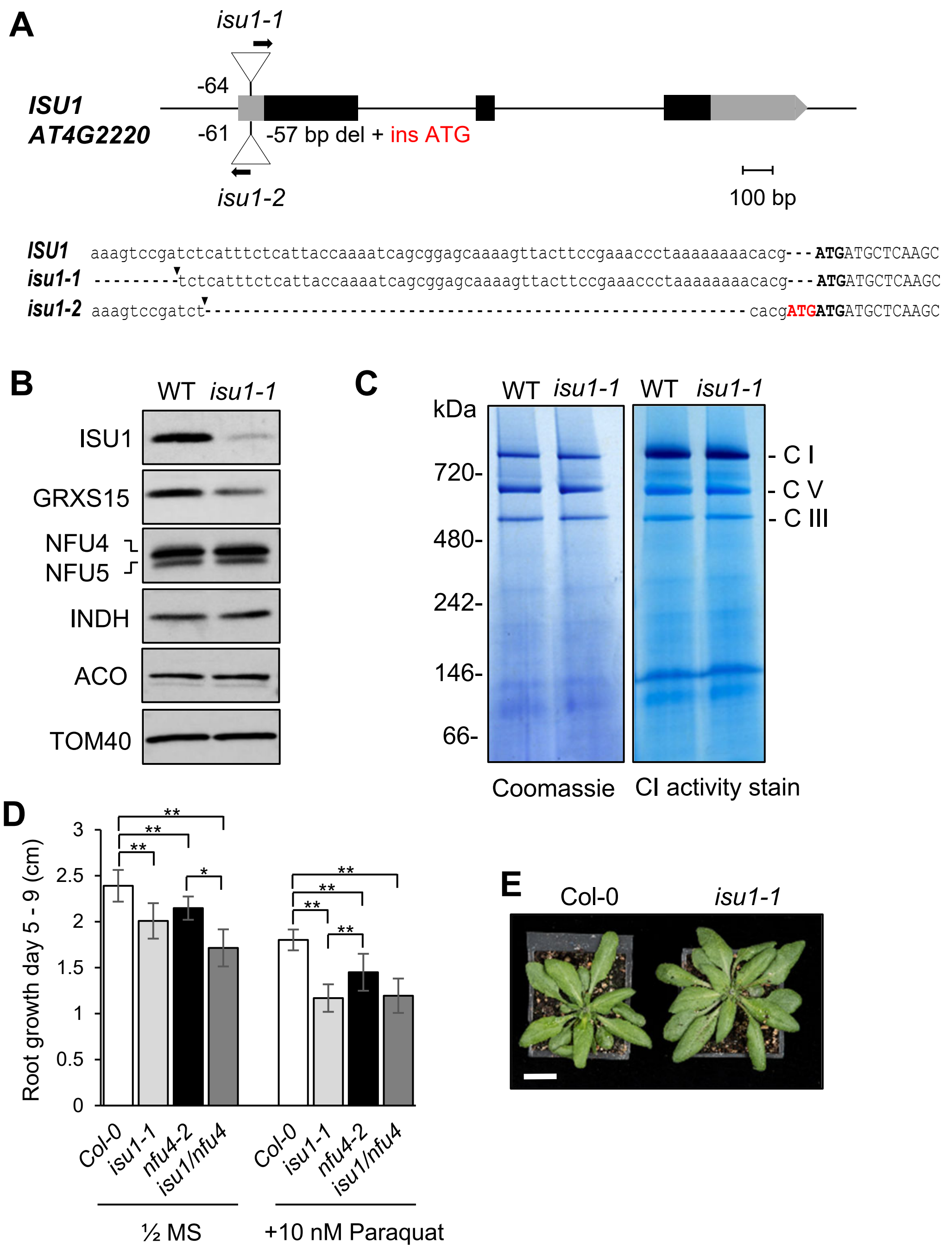

Supplemental Figure S4. Characterization of the *isu1-1* mutant allele alone and combined with *nfu4-2*.
A. Gene model of *ISU1* and the position of T-DNA insertions: *isu1-1*, SALK_006332; *isu1-2*, GK_424D02. Black bars represent exons, grey bars are the 5' and 3' UTRs. Triangles represent T-DNA insertions, an arrow indicates the outward facing left border primer. The numbers mark the nucleotide next to the left-border sequence, relative to the ATG start codon.
B. Protein blot analysis of mitochondria from cell culture, wild type (WT) and *isu1-1*, using antibodies against the indicated proteins. GRXS15, glutaredoxin S15; INDH, Iron-sulfur protein required for NADH Dehydrogenase Homologue; ACO, aconitase; TOM40, translocase of the outer membrane protein of 40 kDa.
C. Blue-native PAGE of mitochondria from cell culture, stained with Coomassie (left), or with NADH and nitroblue tetrazolium to visualize complex I activity (right).
D. Root elongation in seedlings grown on vertical agar plates. Seedlings were grown on half-strength Murashige & Skoog (½MS) medium for 5 days, then transferred to fresh plates without or with 10 nM Paraquat, an inducer of reactive oxygen species in mitochondria, and grown for another 4 days. Values represent the average elongation from day 5 to day 9, error bars represent SD. *p < 0.01, **p < 0.001 (Student t-test) , n=30-31 for Col-0; n=13-14 for *isu1-1;* n=11 for *nfu4-2* on ½ MS; n=15 for *nfu4-2* on ½ MS + Paraquat*;* n=6 for *isu1-1 nfu4-2* on ½ MS*;* n=8 for *isu1-1 nfu4-2* on ½ MS + Paraquat.
E. Growth phenotype of 4-week-old wild-type and *isu1-1* plants. Scale bar: 1 cm.

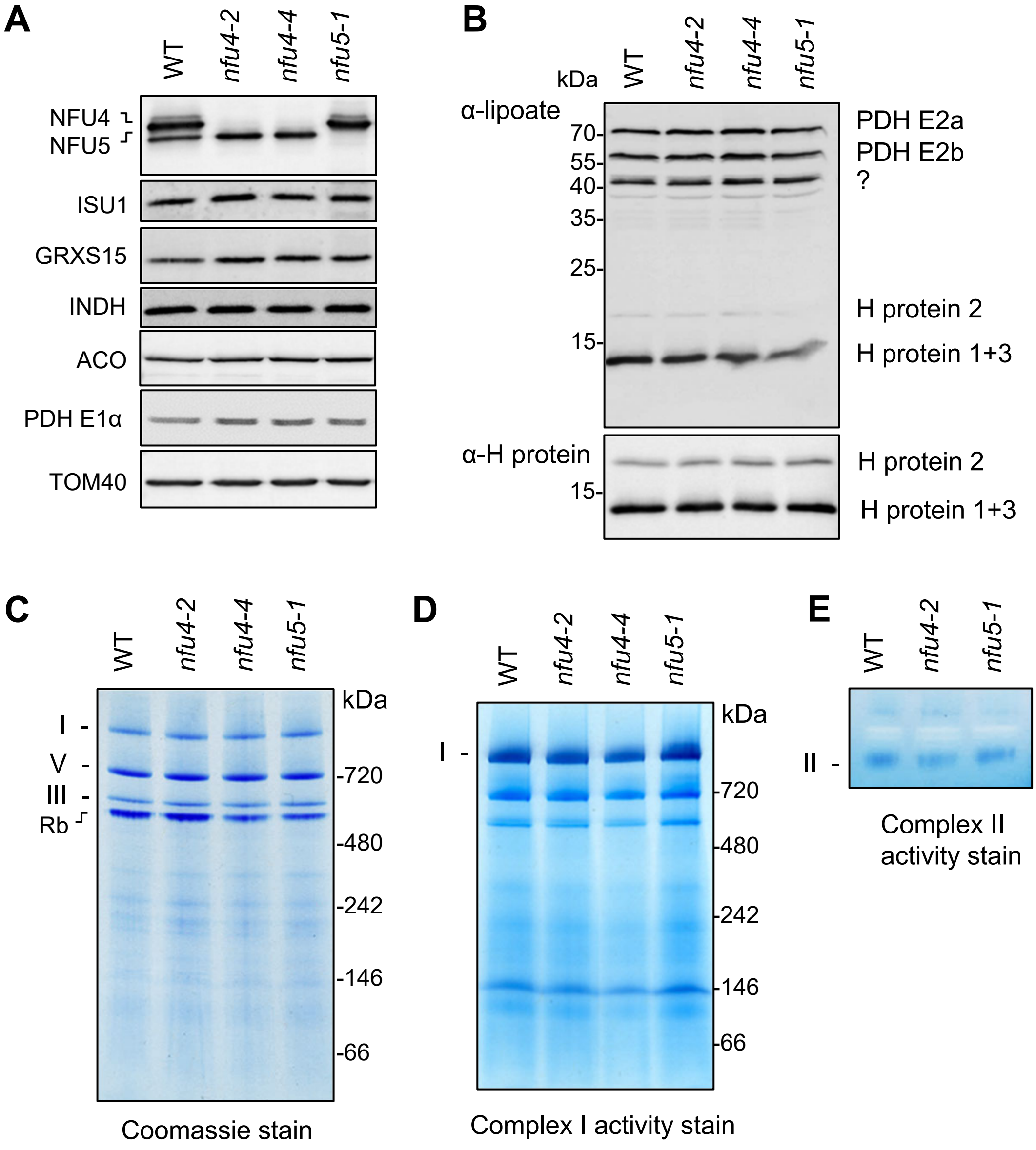

Supplemental Figure S5. Protein blot and BN-PAGE analysis of mitochondria from *nfu4* and *nfu5* single mutants.

A. Protein blot analysis of NFU4, NFU5, aconitase (ACO), Fe-S cluster assembly scaffold ISU1, glutaredoxin S15 (GRXS15), Fe-S protein required for NADH Dehydrogenase homolog (INDH), E1α subunit of pyruvate dehydrogenase (PDH E1α) and translocase of the outer membrane 40 (TOM40). Mitochondria were purified from 2-week-old seedlings of wild type (WT, Col-0), *nfu4* and *nfu5* single mutants, as indicated.

B. Protein blot analysis of lipoate-binding proteins and H proteins of the glycine decarboxylase complex using the same samples as in (A).

C. BN-PAGE of mitochondrial membranes followed by Coomassie staining to visualize respiratory complexes I, III and V. Mitochondria were purified from 2-week-old seedlings. Rb, Rubisco.

D. BN-PAGE of mitochondrial membranes followed by activity staining of respiratory complex I. Mitochondria were purified from callus culture of the indicated lines.

E. As in (D), but after activity staining of respiratory complex II.

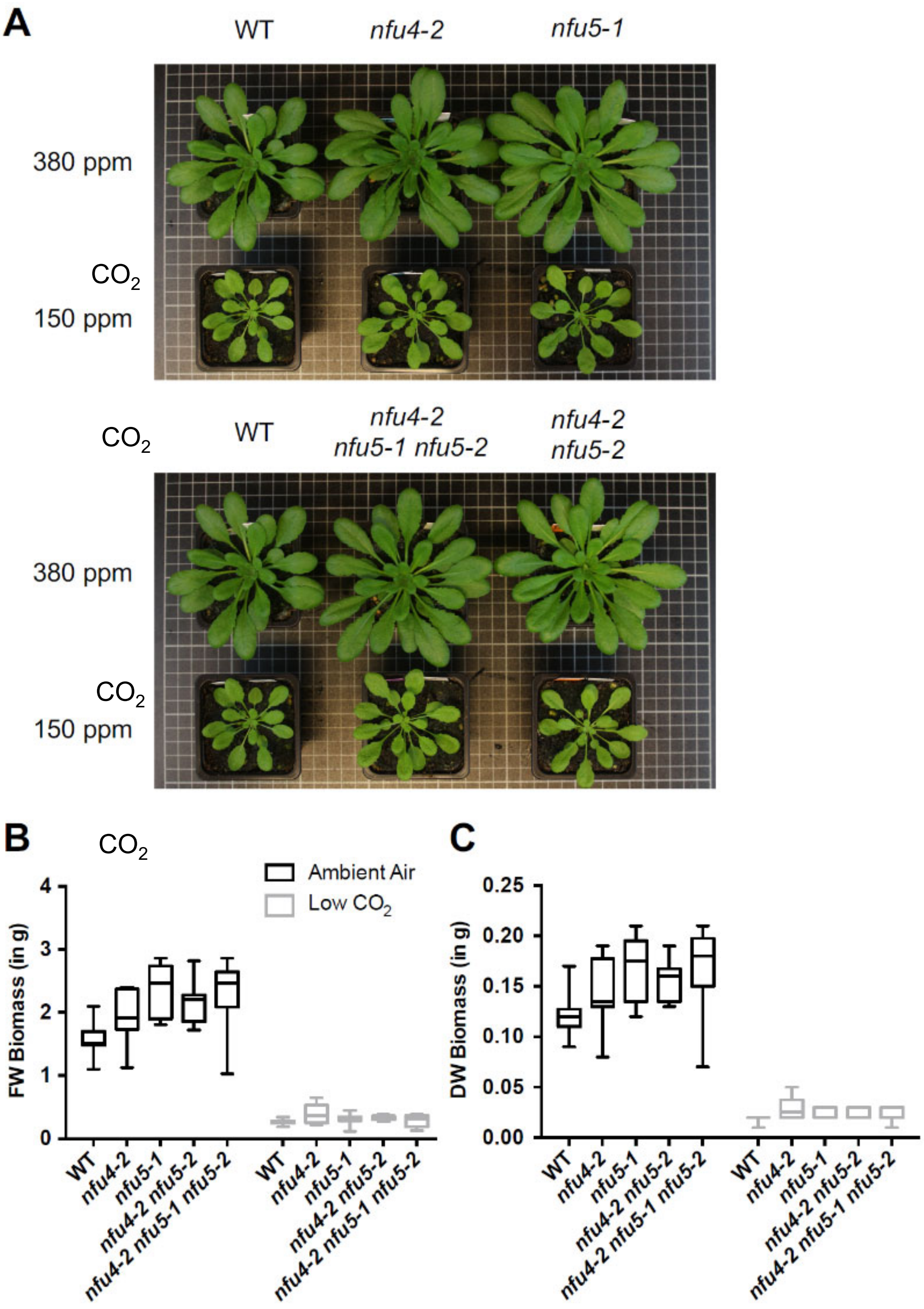

Supplemental Figure S6. Growth of *nfu4-2 nfu5* mutants under low CO_2.
A. Seven-week-old plants grown for 5 weeks in either ambient air containing 380 ppm CO_2 or in 150 ppm CO_2. Other growth conditions were: 8/16h light/dark cycle, 22/17 °C, 75% relative humidity and 180 µmol m^{-2} s^{-1} white light at plant level.
B, C. Fresh weight (FW) and dry weight (DW) of aerial parts of plants in (A). n = 8; the interquartile range of the box is from the 25th to 75th percentiles, and the middle line is the median; whiskers represent min and max values.

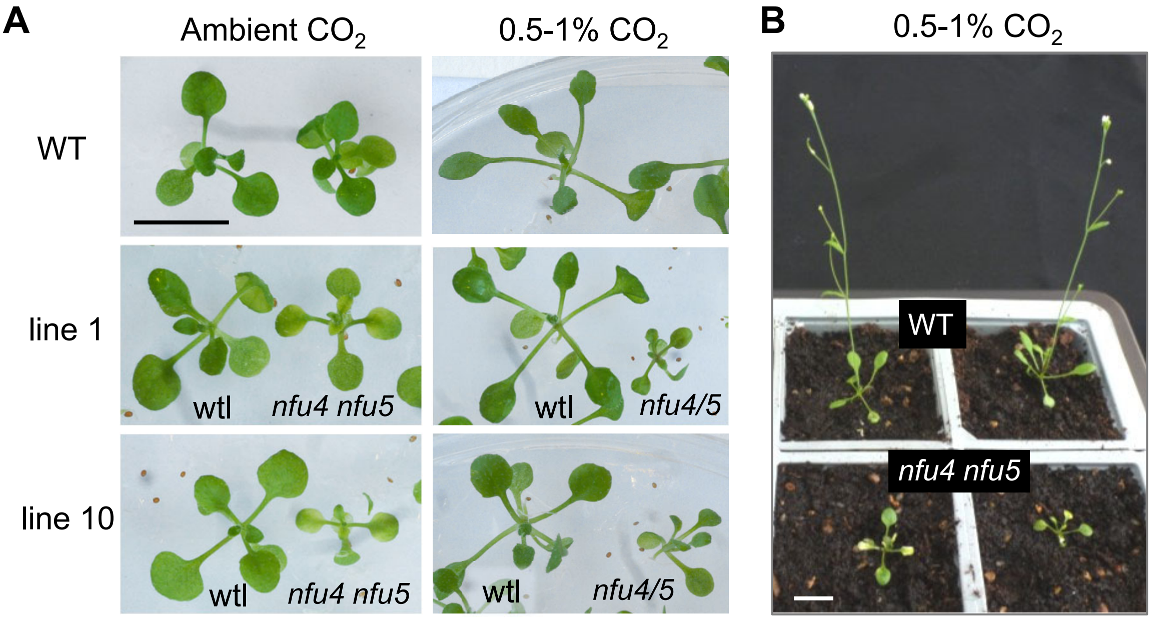

Supplemental Figure S7. Elevated CO_2 cannot rescue growth of *nfu4 nfu5* mutants to produce seeds.
A. Wild-type *A. thaliana* (WT, ecotype Colombia-0) and segregating *nfu4-2*$^{-/-}$ *nfu5-1/nfu5-2* mutant lines (see main text for details) were grown on ½ MS agar medium under ambient CO_2 (~23 ºC) or under elevated CO_2 (0.5 – 1% v/v, ~25 ºC). Images were taken after 14 days, showing a wild-type like (wtl) segregant on the left and a *nfu4 nfu5* mutant seedling on the right. Under high CO_2, mutant seedlings are less chlorotic and produce a second pair of true leaves. Scale bar is 1 cm.
B. WT and mutant seedlings were transferred to soil and photographed after another 8 days. Scale bar is 1 cm.

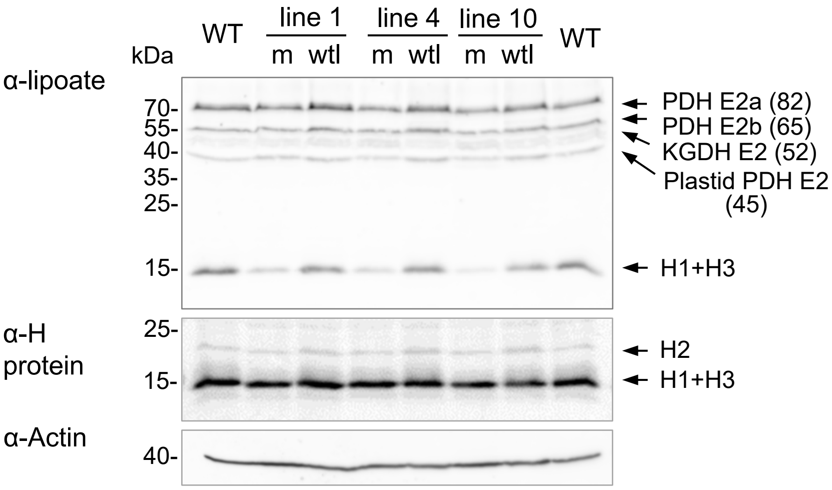

Supplemental Figure S8. Protein blot analysis of lipoylated proteins.
Wild-type (WT), segregating *nfu4-2 nfu5-1* mutant seedlings (m) and their wild-type like siblings (wtl) were subjected to protein blot analysis with antibodies again lipoate and H protein. Actin was labelled to verify equal loading and protein transfer.

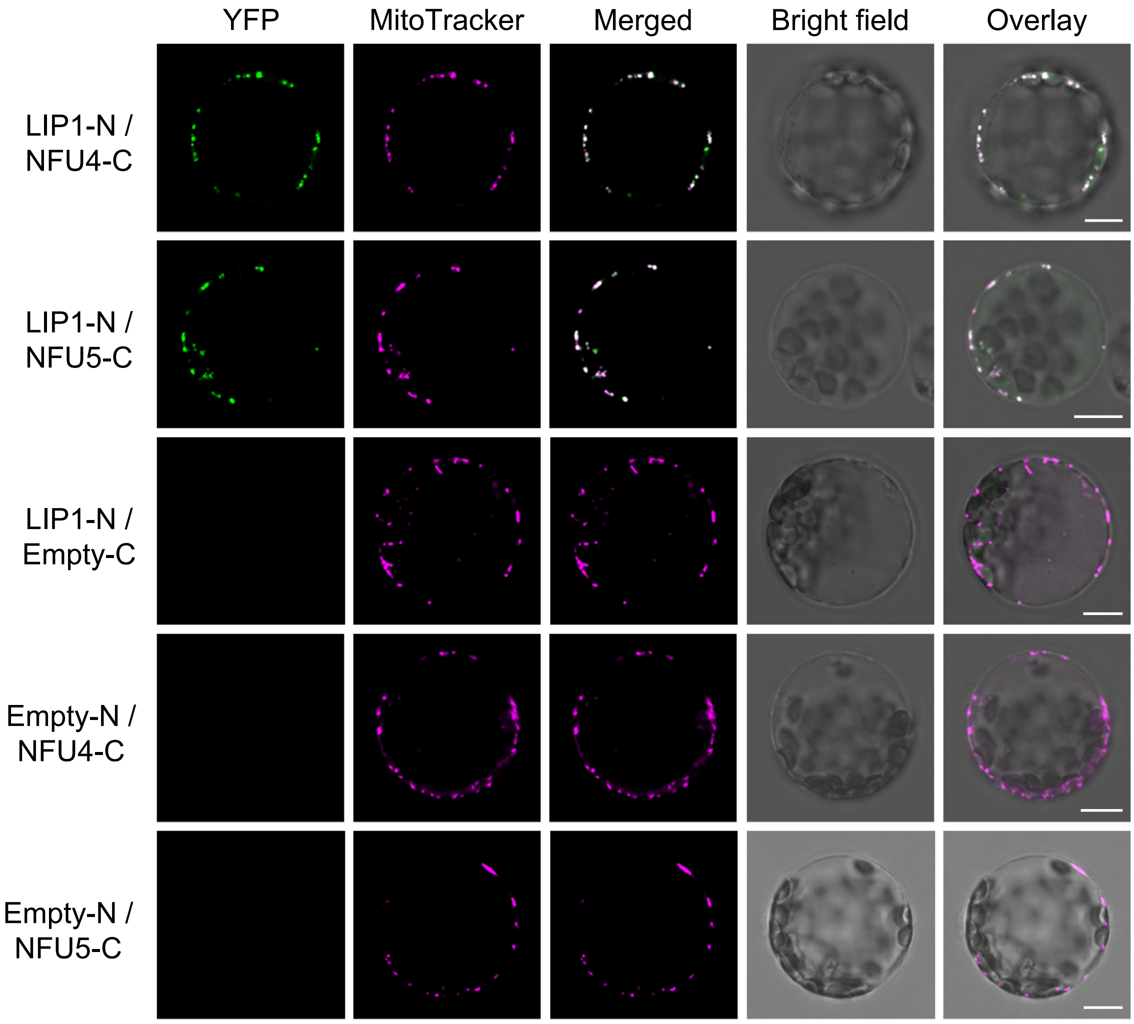

Supplemental Figure S9. Bimolecular fluorescence complementation to test interaction between LIP1 and NFU4/5, continued. Images of the cells presented in Fig. 8 but without maximum Z-stack intensity projection. Because of the strong signal obtained for the interactions between LIP and NFU4 or NFU5, the argon laser was set to its minimum power to limit saturation of the signal, but this setting also reduces the background pixels in the negative control images to near zero. Bars = 10 m.

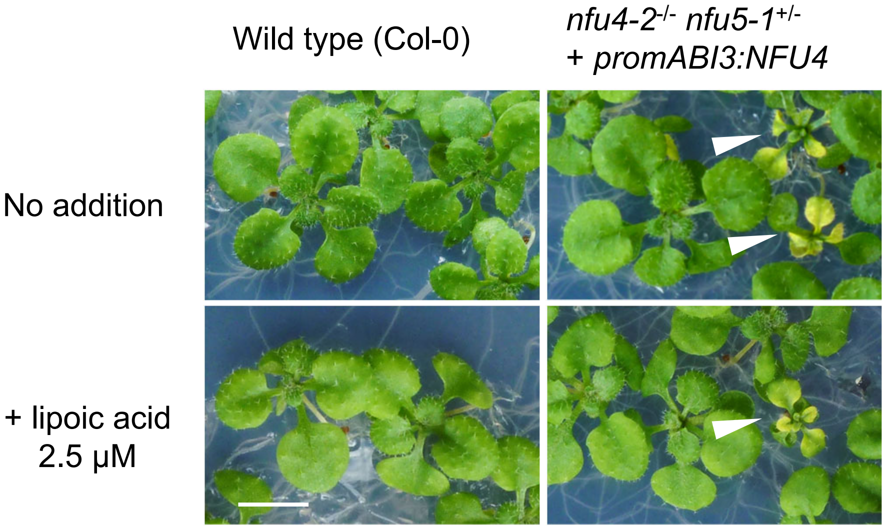

Supplemental Figure S10. Lipoic acid in growth medium does not rescue the *nfu4 nfu5* mutant phenotype. Seeds of wild-type *A. thaliana* and a segregating *nfu4 nfu5* mutant line (see main text for details) were sterilized and grown on ½ MS agar medium with or without 2.5 µM lipoic acid under standard conditions. The images were taken after 18 days. Arrow heads indicate *nfu4 nfu5* double mutant seedlings among normal growing segregants (lacking NFU4 but intact NFU5, see main text). Scale bar is 1 cm.

Supplemental Table S1. *isu1*, *nfu4* and *nfu5* T-DNA insertion lines used in this study.

Locus	Allele	Polymorphism	Mutation
AT4G22220	*isu1-1*	SALK_006332	T-DNA insertion (promoter -65)
(*ISU1*)	*isu1-2*	GK_424D02	T-DNA insertion (promoter -61) ; deletion of 61 nt directly upstream of the ATG.<br><br>No homozygous *isu1-2* mutants could be obtained (genotyping PCR showed 19/19 of sulfadiazine resistant seedlings were heterozygous). Siliques showed ~25% early-stage aborted seed.
AT3G20970	*nfu4-1*	SALK_035493	T-DNA insertion (exon 6 +56; ATG +1774)
(*NFU4*)	*nfu4-2*	SALK_061018	T-DNA insertion (intron 1 +84; ATG +307)
	nfu4-4	SAIL_1233_C08	T-DNA insertion (intron 3 +230; ATG +1011)
AT1G51390	*nfu5-1*	WiscDsLoxHs069_06B	T-DNA insertion (exon 3 +173; ATG +755)
(*NFU5*)	*nfu5-2*	SK24394	T-DNA insertion (promoter -251; ATG -251)
	nfu5-3	GT_3_2834	T-DNA insertion (exon 2 +103; ATG + 436)

Supplemental Table S2. Free, water-soluble amino acid concentrations in *nfu4-2 nfu5-1* seedlings and wild-type like segregants (wtl, *nfu4-2 NFU5*).

**Amino acid**	***nfu4 nfu5***	**Wild-type like segregant** (*nfu4-2 NFU5*)
Alanine	397.8 ± 143.5	50.6 ± 9.2
Arginine	366.5 ± 274.8	139.2 ± 2.2
Asparagine	393.5 ± 86.3	185.8 ± 24.5
Aspartate	41.0 ± 15.0	105.4 ± 38.3
Cysteine	ND	ND
Glutamate	187.5 ± 18.9	199.3 ± 16.4
Glycine	1753.6 ± 192.6 ***	228.9 ± 74.6
Histidine	28.4 ± 4.8	17.0 ± 4.4
Isoleucine	25.3 ± 10.6	6.8 ± 3.7
Leucine	24.7 ± 3.6 *	8.6 ± 4.1
Lysine	27.0 ± 6.5	13.6 ± 5.5
Methionine	4.2 ± 0.7	3.0 ± 0.3
Phenylalanine	32.2 ± 33.0	4.8 ± 2.2
Proline	31.4 ± 7.6	16.6 ± 5.7
Serine	563.3 ± 40.4 ***	216.6 ± 35.1
Threonine	81.5 ± 25.5	43.6 ± 4.5
Tryptophan	6.0 ± 4.7	1.3 ± 0.4
Tyrosine	6.8 ± 1.0	4.0 ± 2.4
Valine	51.0 ± 5.1 **	15.6 ± 5.4

ND: below detection limit.
Values (nmol gFW^{-1}) are the mean of 3 biological replicates ± SD.
*$p < 0.05$, **$p < 0.01$, ***$p < 0.001$ (Student *t*-test)

Supplemental Table S3. Organic acid concentrations in wild-type, *nfu4-2 nfu5-1* and wild-type like segregants (wtl, *nfu4-2 NFU5*).

**Organic acid** (μg gFW^{-1})	**Wild type (Col-0)**	***nfu4-2 nfu5-1***	**Wild-type like segregant** (*nfu4-2 NFU5*)
Citrate	5.39 ± 2.88	2.71 ± 0.99 *	9.92 ± 3.97 *
α-Ketoglutarate	0.22 ± 0.08	3.41 ± 1.72 **	0.36 ± 0.08
Malate	5.68 ± 2.56	4.04 ± 1.37	19.8 ± 3.94 ***
Pyruvate	0.06 ± 0.015	0.13 ± 0.03 ***	0.09 ± 0.01
Succinate	0.93 ± 0.14	2.02 ± 0.46 ***	1.77 ± 0.38 ***

Values (μg gFW^{-1}) are the mean of 4 – 7 biological replicates ± SD.
*$p < 0.05$, **$p < 0.01$, ***$p < 0.001$ (Student *t*-test, pairwise comparison to wild type)

Supplemental Table S4. Primers used in this study.

Oligo-nucleotide	Purpose	Sequence (5'-3')
ISU1 F1<br>ISU1 R1	Genotyping of *isu1-1, isu1-2*	GAACCATCTAAACCGTCCACG<br>CTCCATAGCTTTGCCTTTCACCC
AM76<br>AM77	Genotyping of *nfu4-1*	CCCAGTTGCTTTAATGAGCTG<br>AACTGTACGCCAAACGTGAAC
NFU4-2 F1<br>NFU4-2 R1	Genotyping of *nfu4-2*	GGTCGACGAAATGAAAGGGATTG<br>GGCTAATGGCGAACCCAGAGCAG
AM81<br>AA82	Genotyping of *nfu4-4*	TCTTGATTCTCCAATGGTTGC<br>CAAGCCTGAGATATTTGCAGC
AM84<br>AM85	Genotyping of *nfu5-1*	TTCACCATCGAATTCTTGCTC<br>CGTCGCTCTCTGTTTATCTCG
AM73<br>AM74	Genotyping of *nfu5-2*	TTCTTAAGCTCTGGTCCCTCC<br>CGAGATAAACAGAGAGCGACG
AM101<br>AM102	Genotyping of *nfu5-3*	ACAGATGAACTTGGACAACCG<br>CGTCGCTCTCTGTTTATCTCG
Left border primers:<br><br>LBb1.3<br>LB3_SAIL<br>L4<br>pSKTAIL-L3<br>Ds3-1<br>GK_o8409	PCR detection of T-DNA insert and sequencing for:<br><br>SALK lines (*isu1-1*, *nfu4-2*, -4)<br>SAIL lines (*nfu4-4*)<br>Wisconsin DsLox lines (*nfu5-1*)<br>SK population (*nfu5-2*)<br>JIC Gene Trap line (*nfu3-5*)<br>GABI-kat lines (*isu1-2*)	 <br>ATTTTGCCGATTTCGGAA<br>TAGCATCTGAATTTCATAACCAATCTCGATACAC<br>TGATCCATGTAGATTTCCCGGACATGAAG<br>ATACGACGGATCGTATTTGTCG<br>ACCCGACCGGATCGTATCGGT<br>ATATTGACCATCATACTCAT
Right border primers<br><br>SALK_RB<br>GK_o2588	PCR and sequencing of right border insertion site of:<br>*isu1-1*<br>*isu1-2*	 <br>TGATAGTGACCTTAGGCGACTTTTGAACGC<br>CGCCAGGGTTTTCCCAGTCACGACG
ACT8_F<br>ACT8_R	RT-PCR of *ACTIN8* (*AT1G49240*)	TAAACTAAAGAGACATCGTTTCCA<br>TTTTTATCCGAGTTTGAAGAGGC
AM93<br>AM94	RT-PCR of *NFU5* in *nfu5-3*	AGCTTCCGTAACTTGTTCTCC<br>AGATGTTCCCTCCTCTTCAC
NFU4 Q1<br>NFU4 Q2	RT-qPCR of *NFU4*	GCAGTTCAAGATGATGGTGGG<br>CAGCATGTTTTCGATTCCCG
NFU5F1<br>NFU5R1	RT-qPCR of *NFU5*	GCGTATCCGACCATCAGTCC<br>CCGCTACAAGCTCCTTGCAT
AtNFU4_GatFW<br>AtNFU_GatRV	Cloning of *ABI3prom*:*NFU4* into pH7WG-pABI3	GGGGACAAGTTTGTACAAAAAAGCAGGCTCC**ATG AAAGGGATTGCGAGGC**<br>GGGGACCACTTTGTACAAGAAAGCTGGGT**TTGTT CAAAAATTGAGTCAATTTTTAC**
AtNFU4-*XbaI*-For<br>AtNFU4-*XhoI*-Rev	BiFC analysis: cloning of *NFU4* into pUC-SPYNE/CE	CCCCTCTAGAATGAAAGGGATTGCGAGG<br>CCCCCTCGAGCTCTACTCTCATCTCTCC
AtNFU4-*NcoI*-pCK-For<br>AtNFU4-*BamHI*-pCK-Rev	Localization: cloning of $NFU4_{MTS}$ into pCK-GFP-C65T	CCCCCCATGGCTAAAGGGATTGCGAGGCTTGTA<br>CCCCGGATCCGTTCTTCTCTGCCCTCC
AtNFU5-*NcoI*-pCK-For<br>AtNFU5-*BamHI*-pCK-Rev	Localization: cloning of $NFU5_{MTS}$ into pCK-GFP-C65T	CCCCCCATGGCTAAAGGGCTTACGAGGCTTCTC<br>CCCCGGATCCGTCCTCCTTTGCTCGAT
AtNFU5-*XbaI*-For<br>AtNFU5-*XhoI*-Rev	BiFC analysis: cloning of *NFU5* into pUC-SPYNE/CE	CCCCTCTAGAATGAAAGGGCTTACGAGG<br>CCCCCTCGAGCTCCATTGGACCAGAAGA

AtLIP1-*XbaI*-For<br>AtLIP1-*SalI*-Rev	BiFC analysis: cloning of *LIP1* into pUC-SPYNE/CE	CCCCTCTAGAATGCATTCGCGCTCCGCC<br>CCCCGTCGACCGGGGATGTAGAAGGAGA
AtNFU4-*NcoI*-For<br>AtNFU4-*BamHI*-Rev	Y2H analysis: Cloning of *NFU4* without MTS in pGAD/pGBK	CCCCCATGGCTTTTATCCAAACCCAATCA<br>CCCCGGATCCCTACTCTACTCTCATCTC
AtNFU5-*NdeI*-For<br>AtNFU5-*BamHI*-Rev	Y2H analysis: Cloning of *NFU5* without MTS in pGAD/pGBK	CCCCCCCCATATGTTTATCCAAACACACAA<br>CCCCGGATCCTCACTCCATTGGACCAGA
AtLIP1-*NdeI*-For<br>AtLIP1-*BamHI*-Rev	Y2H analysis: Cloning of *LIP1* without MTS in pGAD/pGBK	CCCCCCCCATATGTTCTCCTCTTCCTCGGCT<br>CCCCGGATCCTTACGGGGATGTAGAAGG
AtBIO2-*NdeI*-For<br>AtBIO2-*BamHI*-Rev	Y2H analysis: Cloning of *BIO2* without MTS in pGAD/pGBK	CCCCCCCCATATGTCTTCATTATCTGCTGCT<br>CCCCGGATCCTTAGTGAGAAGCGGAAGC
AtACO2-*BsmbI*-For<br>AtACO2-*SmaI*-Rev	Y2H analysis: Cloning of *ACO2* without MTS in pGAD/pGBK	CCCCCCCGTCTCCCATGGCTTCTGAGCATTCCTA CA<br>CCCCCCCGGGTTACTTGGCGCTCAAACT
